# Challenging the silent temporomandibular joint paradigm in children with juvenile idiopathic arthritis

**DOI:** 10.1186/s12969-022-00681-y

**Published:** 2022-04-02

**Authors:** Willemijn F. C. de Sonnaville, Michel H. Steenks, Caroline M. Speksnijder, Nico M. Wulffraat, Antoine J. W. P. Rosenberg

**Affiliations:** 1 Department of Oral and Maxillofacial Surgery and Special Dental Care, University Medical Center Utrecht, Utrecht University, Heidelberglaan 100, 3584 CX, PO Box 85500, Utrecht, The Netherlands; 2 Department of Pediatric Rheumatology and Immunology, Wilhelmina Children’s Hospital, University Medical Center Utrecht, Utrecht University, Utrecht, The Netherlands

Dear Sir

We write to address the silent temporomandibular joint (TMJ) paradigm and our attempt to study this phenomenon. According to the silent TMJ paradigm, children with juvenile idiopathic arthritis (JIA) may have TMJ inflammation without any clinical sign or symptom [1]. Not recognizing TMJ inflammation may result in TMJ dysfunction, mandibular growth disturbances and subsequent maxillofacial asymmetries. Early detection and treatment of inflammation may therefore prevent these sequelae. Current diagnostic methods are clinical examination, radiographic imaging, and magnetic resonance imaging (MRI). Contrast enhanced MRI has been accepted as the gold standard for detection of TMJ arthritis. Despite its inherent disadvantages, routine contrast enhanced MRI has been advocated for early diagnosis of TMJ inflammation. In a recent study we aimed to assess the prevalence of ‘the clinical silent TMJ’ in children with new onset, not yet treated JIA and a MRI confirmed TMJ arthritis.

A prospective study in children with new onset JIA was conducted between January 2018 and February 2020 at the outpatient clinic of the Department of Pediatric Immunology and Rheumatology, the University Medical Center (UMC) Utrecht, The Netherlands. The inclusion rate was low and we were not able to achieve the sample size needed to draw conclusions. The study was thus stopped prematurely.

Thirty two children were excluded because of 1) patients not willing to participate (*n*=5); 2) rheumatologist unsure of JIA diagnosis at first examination (*n*=7); 3) treatment, already started due to a delay between the first consultation with the rheumatologist and the presentation to the researcher (*n*=11); 4) patients having orthodontic treatment (*n*=2); 5) the presence of at least one clinical TMJ sign or symptom (*n*=7). A total of 3 children with new onset JIA and 4 children with an exacerbation of JIA, after a period of full clinical remission were included.

None of the seven included children with a TMJ protocol score zero [2], indicating the absence of any clinical TMJ sign or symptom, showed MRI established TMJ inflammation.

Our inclusion rate partly represents the concerns of patients regarding the disadvantages of MRI screening such as the need for intravenous contrast infusion and sedation in case of anxiety, including concerns over contrast retention in the (young) human brain. To overcome these disadvantages, it is in our opinion necessary to assess the value of a comprehensive clinical examination to selectively identify children for further MRI imaging instead of prescribing a routine MRI. The silent TMJ involvement is mostly mentioned in studies comparing only a few clinical TMJ variables to MRI outcomes [3–5]. A combination of clinical variables demonstrated a higher sensitivity for TMJ arthritis than each clinical variable separately [4]. Extending the clinical examination with multiple variables may narrow the indication for MRI of the TMJ [2]. To set out a validated and tailored diagnostic pathway for TMJ arthritis in children with JIA we hope that an organization such as the Temporomandibular Joint Juvenile Arthritis Work group (TMJaw) will be able to follow up on our suggestion to verify or deny the silent TMJ paradigm.

## Data Availability

The datasets used and/or analyzed during the current study are available from the corresponding author on reasonable request.
